# Biokinetic Evaluation of Contrast Media Loaded Carbon Nanotubes Using a Radiographic Device

**DOI:** 10.3390/toxics9120331

**Published:** 2021-12-02

**Authors:** Mieko Takasaka, Shinsuke Kobayashi, Yuki Usui, Hisao Haniu, Shuji Tsuruoka, Kaoru Aoki, Naoto Saito

**Affiliations:** 1 Institute for Biomedical Sciences, Interdisciplinary Cluster for Cutting Edge Research, Shinshu University, 3-1-1 Asahi, Matsumoto 390-8621, Japan; 18hb405k@shinshu-u.ac.jp (M.T.); hhaniu@shinshu-u.ac.jp (H.H.); 2 Biomedical Engineering Division, Graduate School of Science and Technology, Shinshu University, 3-1-1 Asahi, Matsumoto 390-8621, Japan; 3 USUI Orthopedic Clinic, 6-33 Idegawa, Matsumoto 390-0826, Japan; cobber1206@gmail.com (S.K.); yk-us@mvj.biglobe.ne.jp (Y.U.); 4 Institute of Carbon Science and Technology, Interdisciplinary Cluster for Cutting Edge Research, Shinshu University, 4-17-1 Wakasato, Matsumoto 380-8553, Japan; s_tsuruoka@shinshu-u.ac.jp; 5 Physical Therapy Division, School of Health Sciences, Shinshu University, 3-1-1 Asahi, Matsumoto 390-8621, Japan

**Keywords:** carbon nanotube, peapod, biokinetics, biodistribution

## Abstract

Considerable progress has been made in various fields of applied research on the use of carbon nanotubes (CNTs). Because CNTs are fibrous nanomaterials, biosafety of CNTs has been discussed. The biokinetic data of CNTs, such as using the radioisotope of carbon and surface labeling of CNTs, have been reported. However, the use of radioisotopes requires a special facility. In addition, there are problems in the surface labeling of CNTs, including changes in surface properties and labels eliminating over time. In order to solve these problems and properly evaluate the biokinetics of CNTs, the authors synthesize peapods with platinum (Pt) encapsulated within the hollow region of Double-Walled CNTs (DWCNTs) and develop a new system to evaluate biokinetics using widely available imaging equipment. In the cell assay, no significant difference is observed with and without Pt in CNTs. In animal studies, radiography of the lungs of rats that inhaled Pt-peapods show the detectability of Pt inside the CNTs. This new method using Pt-peapods enables image evaluation with a standard radiographic imaging device without changing the surface property of the CNTs and is effective for biokinetics evaluation of CNTs.

## 1. Introduction

Carbon nanotubes (CNTs) have been widely available and commercialized over 10 years [1,2,3]. However, many aspects regarding the biokinetics of CNTs remain unclear [4,5,6]. The toxicity of CNTs has been evaluated with experimental animal models using various methods such as inhalation exposure and intravenous administration [7]. While several studies report the distribution of inhaled CNTs to organs other than the lungs [8,9], others do not suggest their migration to other systemic organs [10]. The differences in biokinetics between reports may have resulted from the fact that the biological effect of CNTs depends on the types, size, functionalization methods, and dispersant [11,12,13,14]. Although biomedical applications of CNTs have been widely researched for potential uses such as a drug delivery system (DDS) [15,16], therapeutic agents that utilize its thermal effects induced by microwaves, light, or radio frequencies [17], and tissue scaffolds for engineering [18]. No products have thus far been put into practical use [4,19]. The reason for the slow commercial implementation is due to the safety of CNTs, which has not yet been clarified.

Previously reported methods for evaluating the biokinetics of CNTs include the preparation and observation of tissue sections [20,21], the use of contrast media as a label to bind on the surface of CNTs [22], the use of 14C radioisotope-labeled CNTs [9,23], Raman spectroscopy of single-walled CNTs (SWCNTs) [24,25], and methods for detecting near-infrared fluorescence [26,27,28,29]. However, there are inherent problems with these methods, including the quantitative limitation of prepared tissues, changes in surface properties, and labels eliminating over time. The use of radioisotopes requires the preparation of radioactive CNTs, for which a special facility has to be used. More recently, a method for detecting near-infrared fluorescence has been developed, which is suitable for detecting SWCNTs; however, the measurement of multi-walled CNTs (MWCNTs) is not possible with this method [28]. Similarly, MWCNTs cannot be detected by Raman spectroscopy.

To solve these technical issues, we have developed a technique to evaluate the biokinetics of CNTs with a diagnostic imaging device by placing contrast media in the hollow structure of CNTs. These CNTs are referred to as “peapods”, as the particles contained within the hollow structure of CNTs resemble peapods [30]. Applying contrast media to the encapsulating CNTs for biokinetic evaluation enables visualization by magnetic resonance imaging (MRI) or radiographic imaging devices, thereby allowing the observation of its biodistribution in vivo. The advantages of this method include the prevention of eliminating contrast media with encapsulating CNTs and the ability to evaluate biokinetics while maintaining the original surface morphology of CNTs. In other words, the newly developed technique allows biokinetic evaluation for commercialized CNTs without surface modification by attaching a particular chemical marker. We successfully produced peapods containing gadolinium (Gd) in 2015 that could be visualized by MRI [31]. In this study, we report the effectiveness of Pt-peapods containing platinum (Pt) by preforming a biokinetic evaluation using radiography with a much higher resolution than MRI.

## 2. Materials and Methods

### 2.1. Pt-Peapod

DWCNTs (Toray DWCNT, Toray, Tokyo, Japan) were washed in pure water and ethanol to remove dispersants and residues, and then heated at 100 °C to remove moisture. Pt-peapods were synthesized using the glassware device shown in Supporting Information Figure S1. A total of 100 mg of DWCNTs were weighed on an electronic balance and placed in the main tube of a two-way Pyrex glass tube, and 100 mg of platinum (II) chloride (PtCl_2_; 166-15821: Wako Pure Chemical Industries, Osaka, Japan) was placed in the side tube of the device. The main tube was heated in a heating mantle at 150 °C and subsequently degassed with a vacuum pump. After an internal pressure of 10^−5^ Pa was reached inside the tube, the neck of the tube was melted and sealed. Both the main and side tubes were placed in a heating mantle at 500 °C for 24 h, cooled to room temperature, and the Pt-peapods were removed from the main tube. The Pt-peapods were first rinsed with weak acid, then ethanol, and finally pure water. After the Pt-peapods were dried overnight at 100 °C, Transmission Electron Microscopy (TEM; JEM-2100 equipped with Cs-corrected unit EM-Z07167T, JOEL), Tokyo, Japan) and X-Ray Fluorescence (XRF) Spectrometry (ZSX Primus II, Rigaku, Tokyo, Japan) were used for evaluating their properties.

### 2.2. Cytotoxicity Test

Human malignant pleural mesothelioma cells (MESO-1; ACC-MESO-1, RCB2292, Riken BRC, Tsukuba, Japan) and human adenocarcinoma cells (A549; RCB0098, Riken BRC, Tsukuba, Japan) were seeded at 10^4^ cells/well in 96-well plates. MESO-1 cells were cultured in RPMI1640 (RPMI1640 with L-Gln, 30264-85: Nacalai tesque, Kyoto, Japan) with 10% fetal bovine serum (FBS; S1820-505: Lot # S10379S1820: Biowest, MO, USA). A549 cells were cultured using Dulbecco’s Modified Eagle Medium (DMEM) 1.0 g/L glucose with L-Gln and sodium pyruvate (8456-65: Nacalai Tesque, Kyoto, Japan) containing 10% FBS. DWCNTs (heated CNTs) that had been vacuum-annealed at 500 °C for 24 h without adding PtCl_2_ were used as controls. We also analyzed the previously reported Gd-peapods for comparison [31]. Pristine CNTs, heated CNTs, Gd-peapods or Pt-peapods were exposed at concentrations of 0.25, 2.5 and 25 µg/mL and cultured for 24 h. A culture medium was used with a dispersant (PS-80; Polysorbate 80 (HX2) ™, NOF, Tokyo, Japan) dissolved at 0.005%, and a sample with this culture medium alone was used as a control. After culturing, alamarBlue Reagent (alamarBlue Cell Viability Reagent, DAL1100: Invitrogen, Carlsbad, CA, USA) was added at 1/10 volume of the culture medium. After 1.5 h, the fluorescence brightness was measured with a fluorescent plate reader (AF2200, Eppendorf, Hamburg, Germany) (Ex/Em = 530/590 nm). The cytotoxicity evaluation was expressed by the ratio of fluorescent intensity of each sample when the brightness of the control was set to 100%, and an analysis of the significant difference between samples at each concentration was performed using the Tukey-Kramer test.

### 2.3. Cellular Uptake Assay

MESO-1 cells and A549 cells were seeded on a 24-well plate at 5 × 10^4^ cells/well. On the day after seeding, the culture medium to which only the dispersant was added was used as a negative control. Pristine CNTs, heated CNTs, Gd-peapods, or Pt-peapods were exposed at concentrations of 2.5 and 25 µg/mL and cultured for 24 h. MESO-1 cells and A549 cells were removed from the culture medium, trypsinized with a 0.5 g/L Trypsin/0.53 mM/L EDTA solution (32778-05: Nacalai tesque, Kyoto, Japan), subsequently washed, and suspended in Dulbecco’s phosphate-buffered saline (DPBS; 21600-044: Thermo Fisher Scientific, Tokyo, Japan) containing 10% FBS. After passing the solution through a mesh with an opening of 67 µm (N-No230T: Sansyo, Tokyo, Japan), the side scatter (SSC) of CNTs incorporated into cells was measured by a flow cytometer (FACSCaliburTM; Becton, Dickinson, NJ, USA). The SSC was then compared by SSC ratio with control cells not exposed to CNTs.

### 2.4. Cytokine Secretion Assay

MESO-1 cells and A549 cells were seeded on a 24-well plate at 5 × 10^4^ cells/well. On the day after seeding, the culture medium to which only the dispersant was added was used as a negative control. Pristine CNTs, heated CNTs, Gd- peapods or Pt-peapods were exposed at concentrations of 2.5 and 25 µg/mL and cultured for 24 h. After centrifugation of the plate, the medium was collected and stored at −80 °C until cytokine measurement. Cytokines were measured using the Human Chemokine Kit (552990: Becton, Dickinson, NJ, America) and Human Inflammation Kit (551811: Becton, Dickinson, NJ, USA), which are cytometric beads assay methods using a flow cytometer. Ten inflammatory cytokines or anti-inflammatory cytokines were measured according to their respective protocols [32,33]: interleukin-8 (IL-8); regulated on activation, normal T cell expressed and secreted (RANTES); monokine induced by interferon-γ (MIG); cytokine chemoattractant protein-1 (MCP-1); interferon-γ–induced protein -10 (IP-10); interleukin-1β (IL-1β); interleukin-6 (IL-6); interleukin-10 (IL-10); interleukin-12p70 (IL-12p70); cytokine necrosis factor (TNF).

### 2.5. Radiography of the Lung Removed after Intratracheal Administration of Pt-Peapods

Intratracheal administration of Pt-peapods was conducted with an emphasis on evaluating the intrapulmonary distribution of CNTs. A suspension was prepared in which Pt-peapods (500 µg/mL) were dispersed in physiological saline containing 0.1% PS-80. Using 7-week old male Wistar rats (Japan SLC, Shizuoka, Japan), the dose of Pt-peapods was defined as the maximum viable amount of the solution in vivo that can be injected from a syringe at 120 µg/animal (600 µg/kg). Rats were subjected to intratracheal administration by inhalation anesthesia with isoflurane and sacrificed 1 week after administration. The lungs were removed after perfusion with saline. The removed lungs underwent formalin fixation, and a radiograph was obtained. A 3D micro X-ray CT (R_mCT, Rigaku, Tokyo, Japan) with a 10 µm/pixel resolution and a 3D X-ray Microscope (Nano3Dx, Rigaku, Tokyo, Japan) with a 270 nm/pixel resolution were used for radiographic imaging. Analysis was performed using the “Analyze Particles” tool of ImageJ [34]. Particles above a certain threshold were detected, and the particle size and number of particles in each lung lobe were measured.

All animal experimentation procedures were carried out in compliance with the guidelines of the Institutional Animal Care Committee of Shinshu University.

## 3. Results

### 3.1. Pt-Peapods

**Figure 1:**
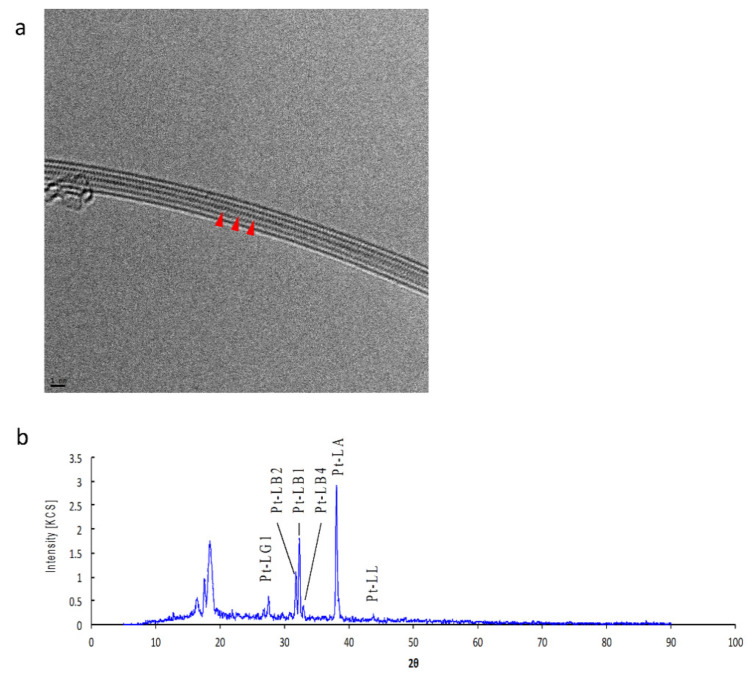
Transmission Electron Microscopy (TEM) image and X-ray fluorescence (XRF) spectrum of platinum(Pt)-peapods: (**a**) In the TEM image, the hollow structure of carbon nanotubes (CNTs) was filled with platinum (II) chloride (PtCl_2_) molecules (red arrow). Scale bar: 1 nm. (**b**) From the XRF spectrum, the Pt content was about 2 wt%, which was approximately the same as the theoretical saturation.

Peapods with encapsulated Pt were prepared. The CNTs used must be open at both ends and the hollow structure of the CNTs must be free of knots. The physical properties of the DWCNTs are described in Supporting Information Table S1. Observation of TEM demonstrated that the hollow space of CNTs was filled with PtCl_2_ molecules (Figure 1). Results from the XRF spectrum showed that the Pt content was approximately 2 wt%, which was approximately the same as the theoretical saturation (Figure 1).

### 3.2. Cell Assay

Cytotoxicity test, cellular uptake assay, and cytokine secretion assay were performed to evaluate the cellular response of the prepared Pt-peapods. MESO-1 and A549 cells were used as lung-associated cells.

**Figure 2:**
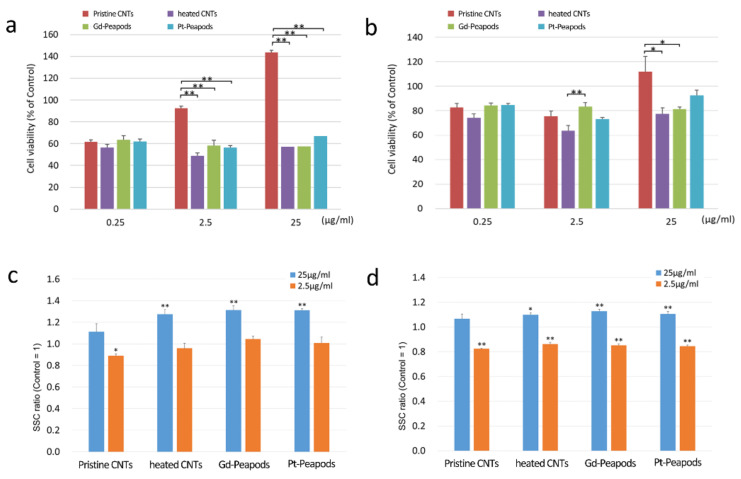
Heated CNTs and Pt-peapods showed similar cell responsiveness in cell assay for lung-related cells, human malignant pleural mesothelioma cells (MESO-1) and human adenocarcinoma cells (A549). (**a**,**b**) Cytotoxicity test. (**a**) MESO-1 cells. (**b**) A549 cells. Mean ± S.E. (*n* = 6). (**c**,**d**) Cellular uptake assay. (**c**) MESO-1 cells. (**d**) A549 cells. Mean ± S.E. (*n* = 4). * *p* < 0.05, ** *p* < 0.01.

In the cytotoxicity test, both MESO-1 and A549 cells showed a significant difference at some concentrations between the non-heat-treated Pristine CNTs and the heat-treated group (heated CNTs, Gd-peapods, Pt-peapods). On the other hand, in the heat-treated group, there was no difference in cytotoxicity at each concentration in both cells except for one. There was a difference in cell viability between cells, and MESO-1 cells showed lower values than that of A549 cells. In addition, the cell viability of each cell did not correlate with the CNT concentration (Figure 2). In the cellular uptake assay of MESO-1 cells, the SSC ratio of the heat-treated group was significantly increased under high-concentration exposure compared with the control, and only the SSC ratio of Pristine CNTs showed a significant decrease under low-concentration exposure. On the other hand, similarly to MESO-1 cells, A549 cells showed a significantly increased SSC ratio in the heat-treated group under high concentration exposure compared to the control; however, the SSC ratio was significantly lower than the control in all groups under low concentration exposure (Figure 2).

**Figure 3:**
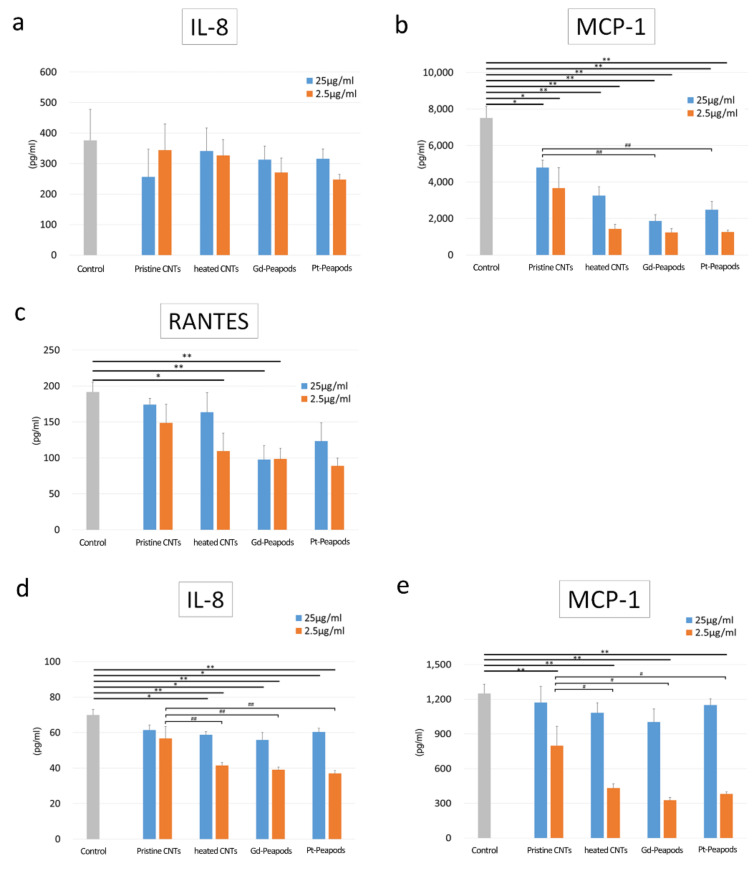
Cytokine secretion assay for lung-related cells MESO-1 and A549: (**a**–**c**) MESO-1 cells. (**d**,**e**) A549 cells. Mean ± S.E. (*n* = 4). * *p* < 0.05, ** *p* < 0.01 (Compared to control), # *p* < 0.05, ## *p* < 0.01 (Compared to each concentration).

Cytokine secretion assay was able to detect the secretion of IL-8, RANTES, and MCP-1, which are known to be produced by a stimulation of inflammatory cytokines in MESO-1 cells; in addition, secretion of IL-8 and MCP-1 was detectable in A549 cells. In MESO-1 cells, there was no difference in the amount of IL-8 secreted by CNTs, but RANTES was significantly decreased in some of the heat-treated group compared to the control, and there was a significant decrease in MCP-1 in the heat-treated group. Furthermore, both Gd-peapods and Pt-peapods showed a significant decrease in MCP-1 compared to Pristine CNTs under high-concentration exposure. On the other hand, in A549 cells, IL-8 was significantly decreased except for Pristine CNTs compared to the control, and a significant difference was observed between the Pristine CNTs and the heat-treated group in the low-concentration exposure group. The same phenomenon was observed for MCP-1. However, a comparison between the MCP-1 control and other groups demonstrated a decrease in MCP-1 secretion only in the low-concentration exposure group, including Pristine CNTs (Figure 3). Other cytokines were below the detection threshold in both the control and exposure groups.

The above results suggest that Pristine CNTs in the heat-treated group may show a different cell responsiveness, and heat treatment may have potentially caused changes in the physical properties of the CNTs. No data from previous results have suggested that Gd or Pt loaded inside the hollow space of CNTs affect cell responsiveness. Therefore, we clarified that heated CNTs and Pt-peapods showed no significant difference in the cytotoxicity test, cellular uptake assay, and cytokine secretion assay, demonstrating a comparable cell responsiveness.

### 3.3. Radiography of the Lung Removed after Intratracheal Administration of Pt-Peapods

**Figure 4:**
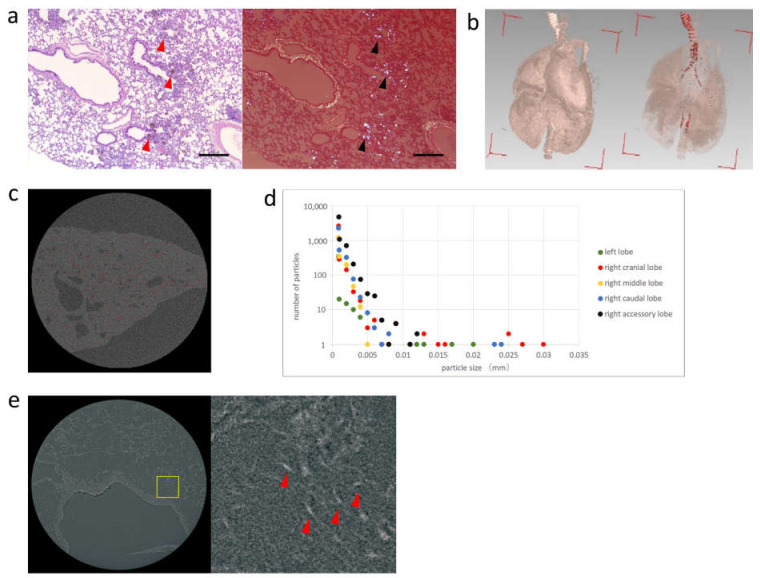
Histological image of the removed lung and X-ray image: (**a**) Light microscopy (left) and polarization microscopy (right) at 1 week after intratracheal administration of Pt-peapods. Pt-peapods were deposited from the main bronchus to the peripheral bronchi. Hematoxylin-Eosin Stain. Scale bar: 300 μm. (**b**) Diagrams for 3D configuration (left) and high absorber detection (right) of 3D micro X-ray CT scanning. A large number of highly absorptive substances in the lung (red dot: minimum of approximately 100 µm^3^) were confirmed from around the tracheal bifurcation to the pulmonary lobe. (**c**) Red point indicates the point of measurement in the 3D micro X-ray CT image of the right lobe. (**d**) The number of particles in each pulmonary lobe after 1 week of intratracheal administration. (**e**) An image (left) of a tissue section of the lung at 1 week after administration of Pt-peapods taken by 3D X-ray microscopy, and an enlarged image in the yellow frame. Pulmonary alveolus was visualized and Pt-peapods as small as approximately 1 µm could be confirmed.

Each pulmonary lobe underwent 3D micro X-ray CT to evaluate the spatial distribution of peapods in the lung. Furthermore, additional tissue sections were taken to perform a detailed evaluation of the intrapulmonary distribution using 3D X-ray microscopy. In addition, tissue specimens were prepared, and the deposition of Pt-peapods was confirmed by light microscopy and polarization microscopy. Pt-peapods were deposited from the main bronchus to the peripheral bronchi (Figure 4).

Three-dimensional analysis software (VGStudio MAX, Nihon Visual Science, Tokyo) was used to produce 3D micro X-ray CT images of lungs removed 1 week after administration. After contrast adjustment, high absorbers indicating Pt-peapods could be identified (Figure 4). The measurements and imaging results of high absorbers as shown in Figure 4 demonstrates that the detected particles from measurements were mostly distributed in the right lobe [34]. In particular, the right cranial lobe was found to have a large particle size. In addition, particle size was confirmed to have a tendency to decrease within the right lobe as the location became more caudal.

Imaging was performed using a tissue section of the lung, which were cut into 2 mm squares, to which Pt-peapods were administered. We previously conducted several studies and optimized methods for lung removal and imaging conditions (Supporting Information Figures S2–S4). The X-ray source was CuKα, and the image was taken at 260 nm/pixel. X-ray absorption imaging enabled the visualization of the structure of pulmonary alveolus and the Pt-peapods that were deposited inside. In the enlarged image, the Pt-peapods presented a needle-like appearance with high absorbers measuring 1.3 µm × 7 µm; thus, for Pt-peapods demonstrating high X-ray absorption, sizes of approximately 1 µm could be confirmed (Figure 4).

## 4. Discussion

One of the reasons for the large discrepancy between reports on the biokinetics of CNTs may occur due to functionalization, such as the binding of contrast media to the surface of CNTs for evaluation [35,36]. Since CNTs have an extremely large specific surface area, the reaction in vivo may differ greatly depending on the molecule that is bound to the CNT surface and alters biokinetic characteristics by the functionalization. In addition, a considerable amount of labeled compounds are eliminated in the body unless they are covalently bonded. On the other hand, when the labeled compounds are covalently bonded, the surface texture of the measured CNTs changes significantly [28,37]. It is possible that the functional molecules and labeled compounds bound to the surface of CNTs may affect each other. In order to evaluate the biokinetics of CNTs, a method of preparing CNTs using carbon radioisotopes and measuring the radiation dose to evaluate the biodistribution has been previously reported [9,23]. Although this method has provided a substantial body of knowledge about biokinetics of CNTs, it cannot be performed in general research facilities because of its requirement of a special facility for handling radioactive substances. In addition, it is not suitable for biokinetic evaluation of commercial CNTs, because the manufacturing process of CNTs must be changed.

For SWCNTs, the Raman spectroscopy method was reported by Liu et al. in 2008, which can be evaluated without the use of contrast media [28]. In recent years, methods for measuring near-infrared fluorescence using the optical properties of SWCNTs have been exhibited [38,39,40]. However, these methods cannot be used for MWCNTs, which are produced in an overwhelmingly large volume compared to SWCNTs. Since MWCNTs are actually used in industrial products, a greater focus has been placed on the evaluation of intra-body migration as they are likely to be inhaled by the human body. In addition, MWCNTs are mainstream for applied research on biomaterials. Therefore, there is a need for a method that can visualize CNTs without surface enhancements or dependence on their optical properties.

To solve these problems, we developed a technique for evaluating the biokinetics of CNTs using peapods, in which labeled compounds are encapsulated in the hollow structure of MWCNTs. These peapods do not change the surface characteristics of CNTs and the labeled compounds do not seep out from the hollow part of CNTs; thus, they are suitable for biokinetics evaluation of MWCNTs. In 2015, we described an MRI evaluation method for peapods containing gadolinium (Gd) [31]. After continued improvement, however, this method was unable to obtain sufficient resolution. In the present report, we attempted to perform evaluations with an X-ray imaging device using Pt-peapods that are easily produced by simply mixing CNTs and Pt, and heating them in a vacuum. The X-ray imaging device provides an overwhelming advantage for the biokinetic evaluation of CNTs, as the device has a much higher resolution than MRI and a high image processing capability that includes 3D rendering.

In the present report, we used DWCNTs, a kind of MWCNTs, with surface characteristics that are most likely to be affected by the encapsulated contrast media. The results of the cell assay showed that Pt-peapods were comparable to the original DWCNTs in all of the cytotoxicity test, cellular uptake assay and cytokine secretion assay. The inclusion of Pt had no biological effect, and this suggests that MWCNTs other than DWCNTs may not alter the biological reaction of the surface. This technique can be used with MWCNTs that are open at both ends and have no junction within the structure.

In animal experiments, Pt-peapods were evaluated using two radiographic devices for the biokinetic assessment of lung tissues, which are the most important tissues for the inhalation of CNTs. Using 3D micro X-ray CT, we were able to obtain a rough evaluation of the spatial distribution of Pt-peapods throughout the lung. However, the resolution was 10 µm/pixel, and Pt-peapods with a small particle size could not be visualized. On the other hand, 3D X-ray microscopy cannot evaluate the entire lung and it must be cut into approximately 2 mm squares. However, its resolution was much higher at 270 nm/pixel, and it was possible to depict the detailed structures of the pulmonary alveolus and deposited Pt-peapods. These structures could be confirmed even with Pt-peapods of approximately 1 µm, which was sufficiently durable for practical use. In biokinetic evaluation, it is effective to evaluate the entire image of one organ by 3D micro X-ray CT scanning, slicing a specimen of the necessary part, and performing a detailed evaluation by 3D X-ray microscopy. The depicted particles are considered to be secondary particles in which primary particles are aggregated based on their size.

As mentioned above, there are many advantages to using Pt-peapods to evaluate the biokinetics of CNTs. Since the outer surface of CNTs is unaltered, it can be evaluated without changing its biological reaction. Moreover, the encapsulated Pt does not come out, which enables an accurate evaluation in order to obtain detailed 3-D images quantitatively with a high-resolution X-ray device. The number of products with industrial applications of CNTs are expected to increase in the future, and the evaluation of biokinetics for inhaled CNTs will become important. Furthermore, as CNTs are used in biomaterials such as DDS, it will be necessary to evaluate the biokinetics of CNTs with various chemical modifications. With the widespread use of CNTs, biokinetics evaluation systems using easily manufactured Pt-peapods and simple, high-resolution X-ray devices can be highly effective in clarifying the safety of various CNTs.

## 5. Conclusions

In this study, we developed a method to evaluate the biokinetics of CNTs using Pt-peapods. Pt-peapod can be easily produced by simply mixing CNT and Pt, and heating them in a vacuum. Pt-peapods have been shown to be comparable to the original CNTs in all cell assays. In addition, we were able to obtain detailed 3D images with a high-resolution X-ray device in animal experiments. Therefore, Pt-peapods are suitable for evaluating the biokinetics of CNTs. In the future, the number of products for industrial use of CNTs is expected to increase, and the evaluation of the biokinetics of inhaled CNTs will be of growing importance. Since Pt-peapods can be easily manufactured without modifying the surface, they can be expected to be used in a variety of CNT products.

## Data Availability

Not applicable.

## Supplementary Materials

The following are available online at https://www.mdpi.com/article/10.3390/toxics9120331/s1: Supporting Information Figure S1: Schematic diagram of peapod synthesizing device; Figure S2: The slicing position of the lung for the preparation of histopathological tissue specimen preparation; Figure S3: Optimization of method for lung removal; Figure S4: Optimization of imaging conditions; Table S1: Material characteristics of DWCNTs. Reference [41] is cited in the Supplementary Materials.
