# Supplementary material for: Biokinetic Evaluation of Contrast Media Loaded Carbon Nanotubes Using a Radiographic Device
Source: Toxics. 2021 Dec 2;9(12):331. doi: 10.3390/toxics9120331 (PMC8705935; doi:10.3390/toxics9120331)
Supplement: Supplementary file 1 [file toxics-09-00331-s001.zip › toxics-1455064-supplementary.pdf]

toxics MDPI

Supplementary Materials: Biokinetic Evaluation of Contrast Media Loaded Carbon Nanotubes Using a Radiographic Device

Mieko Takasaka, Shinsuke Kobayashi, Yuki Usui, Hisao Haniu, Shuji Tsuruoka, Kaoru Aoki and Naoto Saito

Materials and Methods

1. Pt-Peapod

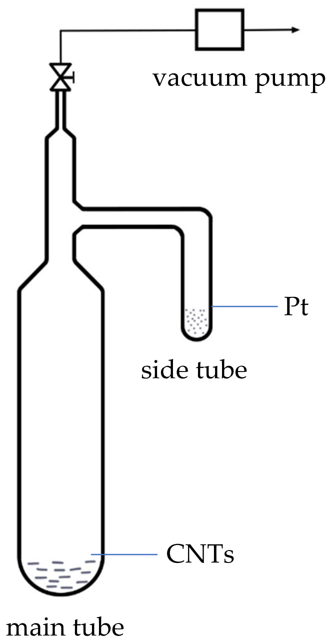

Figure S1. Schematic diagram of peapod synthesizing device.

Table S1. Material characteristics of DWCNTs.

Material	Mean Diameter [nm]	Mean Length [μm]	Purity [%]	Manufacturing Method
DWCNT	< 1.5	not available	> 95	chemical vapor deposition

2. The slicing position of the lung for the preparation of histopathological tissue specimen

This section outlines the histopathological evaluation of the lungs removed after intratracheal administration of Pt-peapods. The histopathological evaluation of the removed lung was performed at a third-party organization, the Japan Bioassay Research Center (Kanagawa, Japan). After intratracheal administration of 120 μg of Pt-peapods, the pulmonary lobes of five animals per evaluation interval were prepared using a previously described slicing method [1] at 1 week, 4 weeks, and 13 weeks after intratracheal administration, as shown in Figure SI 2.

The prepared histopathological specimen was subjected to normal and polarization observation with an optical microscope, to evaluate the deposition of Pt-peapods and the histopathological changes in each organ. The 3D X-ray microscopy images were also compared with the results of the tissue evaluation.

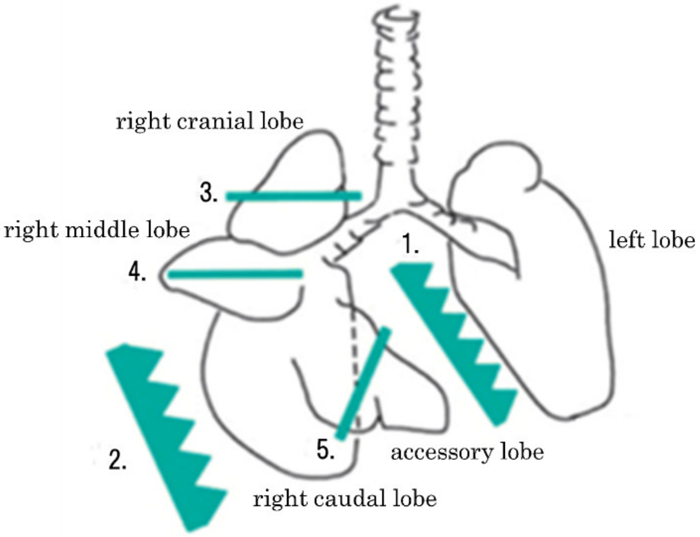

Figure S2. The slicing position of the lung for the preparation of histopathological tissue specimen.

3. Optimization of method for lung removal

In order to produce a simple and optimal method for evaluating specimens, we examined 3D X-ray microscopy images. As a preventative measure, to avoid red blood cells within the specimen from increasing the contrast during imaging, the trachea was ligated in advance, the thorax was expanded, perfusion was performed with physiological saline (300 mL/animal), and the lungs were removed. After formalin fixation, the removed lungs of dried and paraffin-embedded specimens were compared. The removal of red blood cells macroscopically changed the tone of the lung surface to white, and the red blood cells in the lungs were removed, thereby reducing their effect on radiography (Figure SI 3a). Although it was possible to confirm the peapods inside by drying, the luminal structure was difficult to confirm due to collapse. On the other hand, when paraffin was embedded, the luminal structure could be observed (Figure SI 3b). Drying the removed specimen improves the imaging efficiency by reducing the specimen size; however, the luminal structure of the lung remains difficult to confirm. In contrast, although paraffin affects X-ray absorption, paraffin embedding enables the evaluation of the luminal structure by optimizing the conditions of the specimen by removing the red blood cells. Therefore, we decided to perform paraffin embedding (Figure SI 3c).

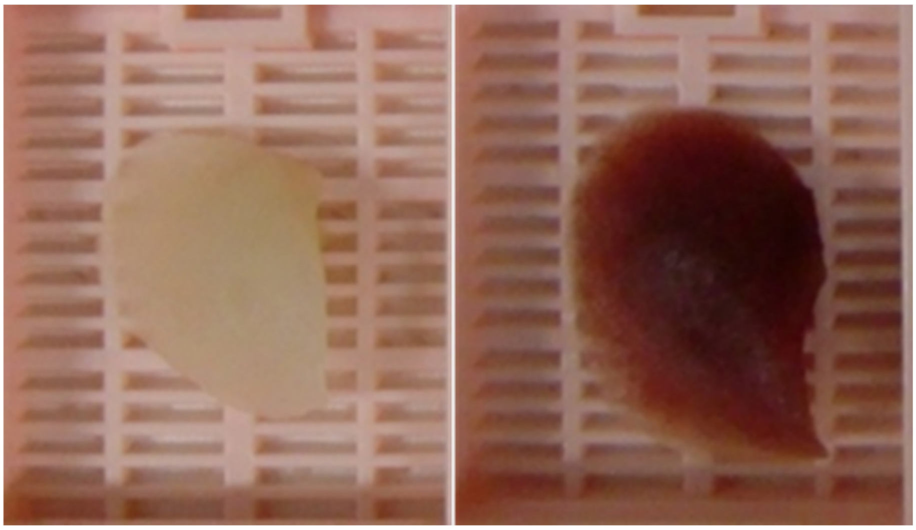

(*a*)

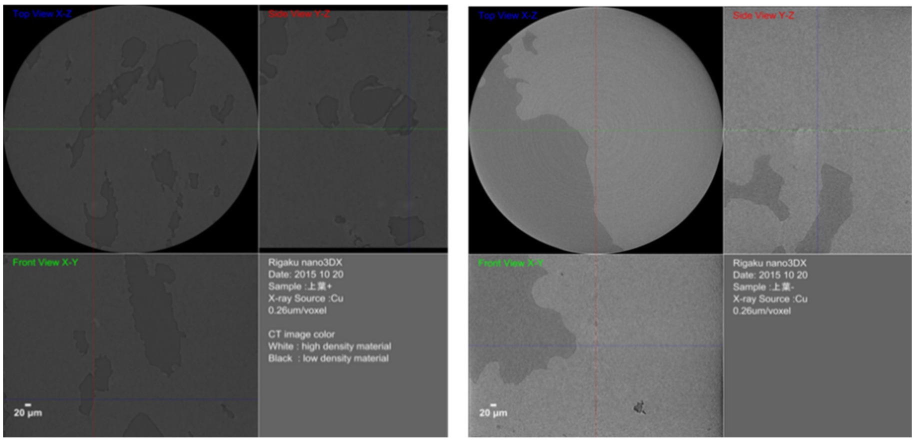

(*b*)

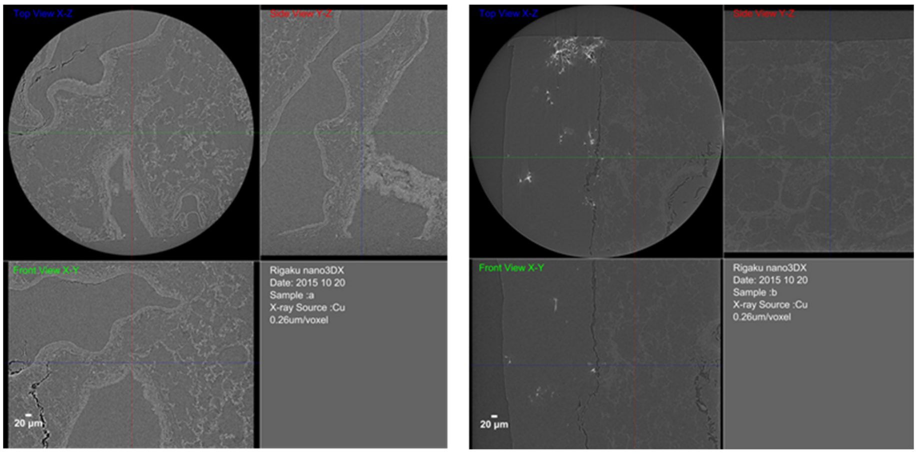

(*c*)

Figure S3. Optimization of method for lung removal. (a) Macroscopic images of lungs after perfusion (left) and lungs without perfusion (right). (b) 3D X-ray microscopy of dried lung tissue. The image on the left shows a dried lung after perfusion to remove as many red blood cells as possible. The image on the right shows a lung after perfusion and formalin fixation. (c) 3D X-ray microscopy of the lung after paraffin embedding after perfusion fixation. The image on the left shows an image of the lung after intratracheal administration of Pt-peapods. The right image shows the lungs after intratracheal administration of pristine CNTs.

4. Optimization of imaging conditions

When the size of the lung tissue was changed and measured, specimens cut into 2 mm squares were determined to be of optimal size. In addition, it was clarified that using

Cu for the X-ray source and an imaging resolution of 260 nm/pixel was most effective when using this specimen. An X-ray absorption image confirmed needle-like appearances. When these were measured, high absorbers could be confirmed, even at a width of approximately 1 μm (Figure SI 4).

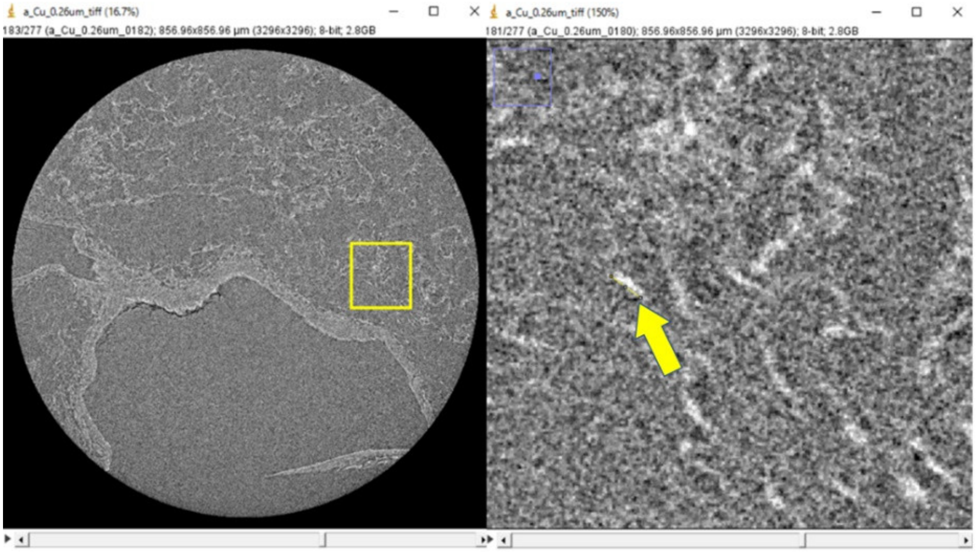

Figure S4. Optimization of imaging conditions.

Cu was used as an X-ray source, and a specimen cut into a 2 mm square was taken at 260 nm/pixel. An X-ray absorption image shows a needle-like appearance with a width of approximately 1 μm. The right figure is an enlarged image of the yellow frame depicted in the left figure. Arrow: X-ray absorption image.

All the experimental protocols were approved by the institutional animal care committee of Shinshu University. The methods were carried out in accordance with the approved guidelines.

References

1. Kittel, B.; Ruehl-Fehlert, C.; Morawietz, G.; Klapwijk, J.; Elwell, M.R.; Lenz, B.; O'Sullivan, M.G.; Roth, D.R.; Wadsworth, P.F. Revised guides for organ sampling and trimming in rats and mice—Part 2. A joint publication of the RITA and NACAD groups. *Exp. Toxicol. Pathol.* **2004**, *55*, 413–431.
